# Muscle Histopathological Abnormalities in a Patient With a CCT5 Mutation Predicted to Affect the Apical Domain of the Chaperonin Subunit

**DOI:** 10.3389/fmolb.2022.887336

**Published:** 2022-06-02

**Authors:** Federica Scalia, Rosario Barone, Francesca Rappa, Antonella Marino Gammazza, Fabrizio Lo Celso, Giosuè Lo Bosco, Giampaolo Barone, Vincenzo Antona, Maria Vadalà, Alessandra Maria Vitale, Giuseppe Donato Mangano, Domenico Amato, Giusy Sentiero, Filippo Macaluso, Kathryn H. Myburgh, Everly Conway de Macario, Alberto J. L. Macario, Mario Giuffrè, Francesco Cappello

**Affiliations:** ^1^ Department of Biomedicine, Neuroscience and Advanced Diagnostics (BIND), University of Palermo, Palermo, Italy; ^2^ Euro-Mediterranean Institute of Science and Technology (IEMEST), Palermo, Italy; ^3^ Department of Physics and Chemistry - Emilio Segrè, University of Palermo, Palermo, Italy; ^4^ Ionic Liquids Laboratory, Institute of Structure of Matter, Italian National Research Council (ISM-CNR), Rome, Italy; ^5^ Department of Mathematics and Computer Science, University of Palermo, Palermo, Italy; ^6^ Department of Biological, Chemical and Pharmaceutical Sciences and Technologies, University of Palermo, Palermo, Italy; ^7^ Department of Health Promotion, Mother and Child Care, Internal Medicine and Medical Specialties, University of Palermo, Palermo, Italy; ^8^ SMART Engineering Solutions & Technologies (SMARTEST) Research Center, eCampus University, Palermo, Italy; ^9^ Department of Physiological Sciences, Stellenbosch University, Stellenbosch, South Africa; ^10^ Department of Microbiology and Immunology, School of Medicine, University of Maryland at Baltimore-Institute of Marine and Environmental Technology (IMET), Baltimore, MD, United States

**Keywords:** CCT5, neurochaperonopathies, chaperonin, neurodegenerative diseases, neuropathies, chaperone system, muscle histopathology, CCT5 apical domain

## Abstract

Recognition of diseases associated with mutations of the chaperone system genes, e.g., chaperonopathies, is on the rise. Hereditary and clinical aspects are established, but the impact of the mutation on the chaperone molecule and the mechanisms underpinning the tissue abnormalities are not. Here, histological features of skeletal muscle from a patient with a severe, early onset, distal motor neuropathy, carrying a mutation on the CCT5 subunit (MUT) were examined in comparison with normal muscle (CTR). The MUT muscle was considerably modified; atrophy of fibers and disruption of the tissue architecture were prominent, with many fibers in apoptosis. CCT5 was diversely present in the sarcolemma, cytoplasm, and nuclei in MUT and in CTR and was also in the extracellular space; it colocalized with CCT1. In MUT, the signal of myosin appeared slightly increased, and actin slightly decreased as compared with CTR. Desmin was considerably delocalized in MUT, appearing with abnormal patterns and in precipitates. Alpha-B-crystallin and Hsp90 occurred at lower signals in MUT than in CTR muscle, appearing also in precipitates with desmin. The abnormal features in MUT may be the consequence of inactivity, malnutrition, denervation, and failure of protein homeostasis. The latter could be at least in part caused by malfunction of the CCT complex with the mutant CCT5 subunit. This is suggested by the results of the *in silico* analyses of the mutant CCT5 molecule, which revealed various abnormalities when compared with the wild-type counterpart, mostly affecting the apical domain and potentially impairing chaperoning functions. Thus, analysis of mutated CCT5 *in vitro* and *in vivo* is anticipated to provide additional insights on subunit involvement in neuromuscular disorders.

## 1 Introduction

Neuromyopathies constitute a large group of diverse diseases typically associated with genetic variants that have a pathogenic impact on nerves and muscles (Morrison 2016; collection of articles in Front Mol Biosci, 2018- Pathologic Conditions of the Human Nervous and Muscular Systems Associated with Mutant Chaperones: Molecular and Mechanistic Aspects). In many of these diseases there is a mutant gene that encodes a component of the chaperone system (CS), e.g., a chaperone gene such as those that code for the chaperonins Hsp60 (Heat shock protein 60) and CCT (Chaperonin Containing TCP1; also called TRiC, TCP1 Ring Complex), and these disorders are classified as neurochaperonopathies (Scalia et al., 2021). An illustrative example of neurochaperonopathy is the distal sensory mutilating neuropathy associated with a point mutation on the CCT5 subunit described earlier (Bouhouche et al., 2006a; Bouhouche et al., 2006b). More recently, we reported a different genetic variant of CCT5 that was associated with a severe distal motor neuropathy (Antona et al., 2020), which is the object of this article.

The hereditary and clinical aspects of chaperonopathies in general, including the neurochaperonopathies, are well established for the most part (Macario and Conway de Macario, 2005; Rubin et al., 2012; Macario et al., 2013). However, the impact of the pathogenic mutation on the properties and functions of the chaperone molecule has been characterized for only very few chaperonopathies, and still less is known on the associated histopathology and the molecular mechanisms responsible for the tissue abnormalities (Macario and Conway de Macario 2020). The scarcity of data on the histopathological manifestations of chaperonopathies impedes advances in the elucidation of the molecular mechanisms underpinning the severe muscular deficiency observed in patients and this, in turn, stands in the way for developing specific treatments. To remedy this lack of necessary knowledge, we studied the available muscle sample from the patient reported earlier, who suffered from a severe motor disorder and carried a point mutation on the CCT5 subunit (Antona et al., 2020). Here, we define histological abnormalities occurring in striated skeletal muscle from this patient. In our *ex vivo* investigation, we asked the few questions that seemed most relevant under the circumstances, pertaining to the status of 1) the muscle tissue; 2) the CCT5 subunit and other pertinent components of the CS; and 3) other molecules involved in the maintenance of the muscle structure and function. Consequently, we performed experiments to reveal the general architecture of the muscle and to determine if it presented signs of atrophy and apoptosis. We also ran tests to determine whether the presence and distribution of the CCT5 subunit were altered in comparison with normal muscle. Likewise, we tested for the presence and location of other components of the CS known to play key roles in skeletal muscle, i.e., alpha-B-crystallin and Hsp90. These are in normal muscle predominantly localized to the Z discs and we looked to see if the muscle from the patient showed this characteristic. We also ran experiments to determine if CCT5 colocalized with another member of the CCT complex, CCT1, which would indicate that the mutant subunit is competent to integrate the chaperoning complex and does form it. We investigated levels of proteins that are known (e.g., actin) or suspected substrates for CCT because any abnormality of these proteins would be an indicator of deficient chaperoning by the chaperonin. Lastly, we tested for the presence and distribution of desmin, the muscle molecule with key functions in the maintenance of overall structure and connections between the main components of the muscle fiber, essential for the coordinated functioning of all of them. The impact of the mutation on the molecular properties of the mutant CCT5 protein were investigated *in silico* by modelling and molecular dynamics simulation.

## 2 Material and Methods

### 2.1 Muscle Tissues and Microscopy

A lateral gastrocnemius skeletal muscle biopsy from the patient, and a corresponding specimen from a healthy individual, were used. The study was approved by the Ethics Committee of University Hospital AUOP Paolo Giaccone of Palermo. Skeletal muscle tissue samples were fixed in 10% buffered formalin and embedded in paraffin. Thin sections (5 µm), obtained from paraffin blocks by microtome, were stained with haematoxylin–eosin and Alcian Blue Pas for histological evaluation. Examination of the sections was performed by two expert pathologists (F. C, and F. R.) with an optical microscope (Microscope Axioscope 5/7 KMAT, Carl Zeiss, Milan, Italy) connected to a digital camera (Microscopy Camera Axiocam 208 color, Carl Zeiss, Milan, Italy).

### 2.2 Immunohistochemistry for Desmin

Sections were obtained from the paraffin blocks of skeletal muscle tissue and dewaxed in xylene for 30 min at 60°C and, after being passed through a descending scale of alcohol concentrations, they were rehydrated in distilled water at 22°C. Subsequently, antigen unmasking was performed with sodium citrate buffer (pH 6) at 75°C for 8 min and then immersion in acetone at −20 °C for 8 min. Then, the sections were immunostained using a Histostain®-Plus third Gen IHC Detection Kit (Life Technologies, Cat. No. 85–9,073). The primary antibody used was anti-desmin mouse monoclonal diluted 1:50 (Table 1). Appropriate negative controls were run concurrently for each reaction. Nuclear counterstaining was performed using hematoxylin (Hematoxylin aqueous formula, REF 05–06012/LN. Cat. No. S2020, Bio-Optica, Milan, Italy). Finally, the sections were examined with an optical microscope as described in the previous Section by two independent observers (F. C, and F. R.), who evaluated the reactions and the immunolocalization on two separate occasions.

**TABLE 1:** Primary antibodies used for immunohistochemistry (IH), and immunofluorescence and double immunofluorescence (IF).

Method	Antigen	Antibody	Supplier	Catalogue Number	Dilution
IF	CCT5	Rabbit polyclonal	Origene	TA308298	1:50
	CCT1 TCP1α (B-3)	Mouse monoclonal	Santa Cruz Biotechnology	SC-374088	
	α-actin	Rabbit polyclonal	Sigma A2066	A2066	
	Myosin heavy chain Type IIX	Mouse monoclonal	DSHB	AB_2266724	
	Alpha-B-crystallin	Rabbit polyclonal	Abcam	AB 5577	
	Hsp90	Rabbit polyclonal	Abcam	AB 13495	
IH-IF	Desmin	Mouse monoclonal	Biocaremedical	CM 036A	

### 2.3 Immunofluorescence

Deparaffinized sections were incubated in the antigen-unmasking solution (10 mM tri-sodium citrate, 0.05% Tween-20) for 10 min at 75 °C, and treated with blocking solution (3% BSA in PBS) for 30 min. Next, the primary antibody (Table 1), was applied, and the sections were incubated in a humidified chamber overnight at 4 °C. Then, the sections were incubated for 1 h at 22°C with a conjugated secondary antibody (anti-rabbit IgG–FITC antibody produced in goat, F0382, Sigma-Aldrich; anti-mouse IgG-TRITC antibody produced in goat, T5393, Sigma-Aldrich). Nuclei were stained with Hoescht Stain Solution (1:1,000, Hoechst 33258, Sigma-Aldrich). The slices on the slides were treated with Perma Fluor Mountant (Thermo Fisher Scientific, Inc. Waltham, MA, United States) and covered with a coverslip. The images were captured using a Leica Confocal Microscope TCS SP8 (Leica Microsystems).

### 2.4 Double Immunofluorescence

Double immunofluorescence was performed as previously described (Marino Gammazza et al., 2014). Briefly, slices of the muscle tissues were deparaffinized, incubated in the antigen unmasking solution for 10 min at 75°C, and treated with blocking solution for 30 min. The sections were then incubated with the first primary antibody (Table 1) overnight at 4°C. The day after, the sections were incubated with the second primary antibody (Table 1) overnight in a humidified chamber at 4°C. Afterwards, the tissue slices were incubated with fluorescent secondary mouse IgG antibody TRITC conjugated (Sigma-Aldrich), diluted 1:250, for 1 h at 22°C, and with FITC conjugated rabbit IgG secondary antibody (Sigma-Aldrich), diluted 1:250, for 1 h at 22°C in a moist chamber. The nuclei were counterstained with Hoechst 33342 for 15 min at 22°C. Finally, the slices were covered with two drops of PBS and mounted with coverslips, using a drop of Vectashield. Imaging was immediately performed with a Leica Confocal Microscope TCS SP8 and the fibers’ positivity for both markers (“merge”) was assessed using the Leica application suite advanced fluorescences software.

### 2.5 In Silico Analyses

We used the structure of crystallized protein deposited in the Protein Data Bank with accession codes 5UYZ (Pereira et al., 2017) to compare the structure of wild type and mutant CCT5 subunit. Study and alignment of CCT5 wild type and mutant linear amino acid sequences was conducted using Clustal Omega multiple sequence alignment program.

### 2.6 Molecular Modelling and Dynamic Simulations

The molecular modelling of the mutant CCT5 subunit was obtained by replacing the amino acid residue Leucine in position 224 with a Valine, using the package Maestro Schrödinger LLC, New York, NY, 2018, version 11.6.010] and compared with the model of the wild type molecule. Molecular Dynamics simulations were performed for 150 ns (in some cases were extended to 200 ns), using the GROMACS 5.1.1 package (Van Der Spoel et al., 2005; Hess et al., 2008). Interactions were described using an all-atoms CHARMM27 force field (Foloppe and MacKerell, 2000; MacKerell and Banavali, 2000). All the simulations for the various systems, and radius of gyration and RMSD analyses were performed as previously described (Antona et al., 2020).

## 3 Results

### 3.1 Atrophy of Muscle Fibers and Disruption of the Tissue Architecture

**FIGURE 1:**
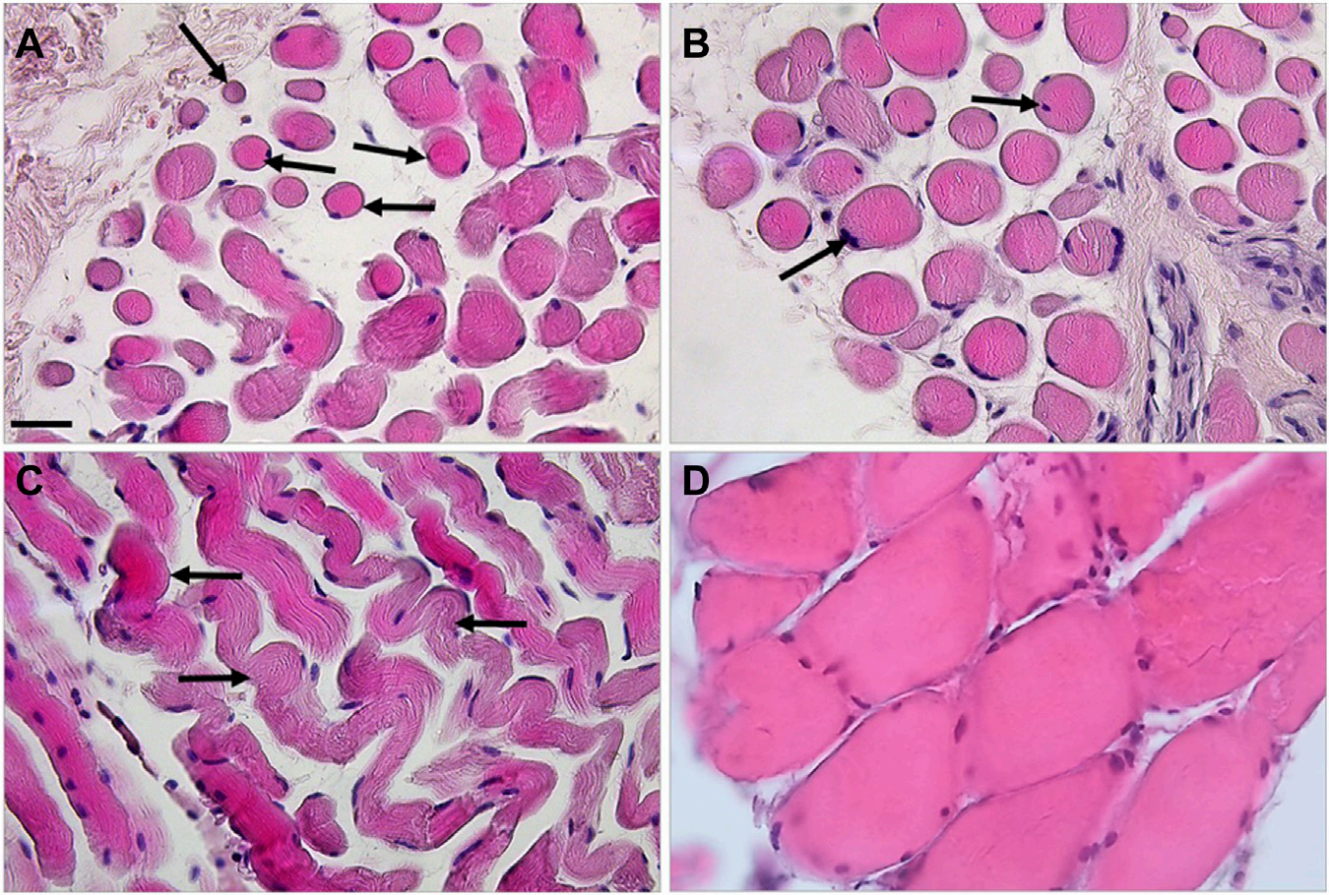
Haematoxylin-eosin staining revealed striking differences between the MUT (panels **A–C**) and CTR (panel **D**) muscles. In cross-section the MUT muscle fibers appeared rounded, of diverse sizes, with swelled nuclei in contact with the sarcolemma or near it (examples shown by arrows on panels **A,B**). These fibers in longitudinal section showed a wavy shape with a striated pattern inside (examples shown by arrows on panel **C**). The inter-fiber space is considerably wider in the MUT than in the CTR muscle. Bar = 100 µm.

Histological analysis showed changes in muscle from the patient (henceforth designated MUT muscle) compared to muscle from the healthy control (henceforth designated CTR muscle). MUT muscle showed widespread atrophy, hypereosinophilia, and disruption of the tissue architecture (Figures 1A–C; Supplementary Figurs S1). The MUT muscle fibers were different in shape and size from those in CTR muscle being predominantly small and rounded in cross section (Figures 1A, B). The same features were revealed with Alcian-Pas staining (Supplementary Figurs S1). In longitudinal sections the MUT muscle fibers had a wavy shape (Figure 1C). In MUT muscle, nuclei were swelled and in contact with the sarcolemma but in some fibers the nuclei were internal, paracentric.

### 3.2 Apoptosis of Muscle Fibers

**FIGURE 2:**
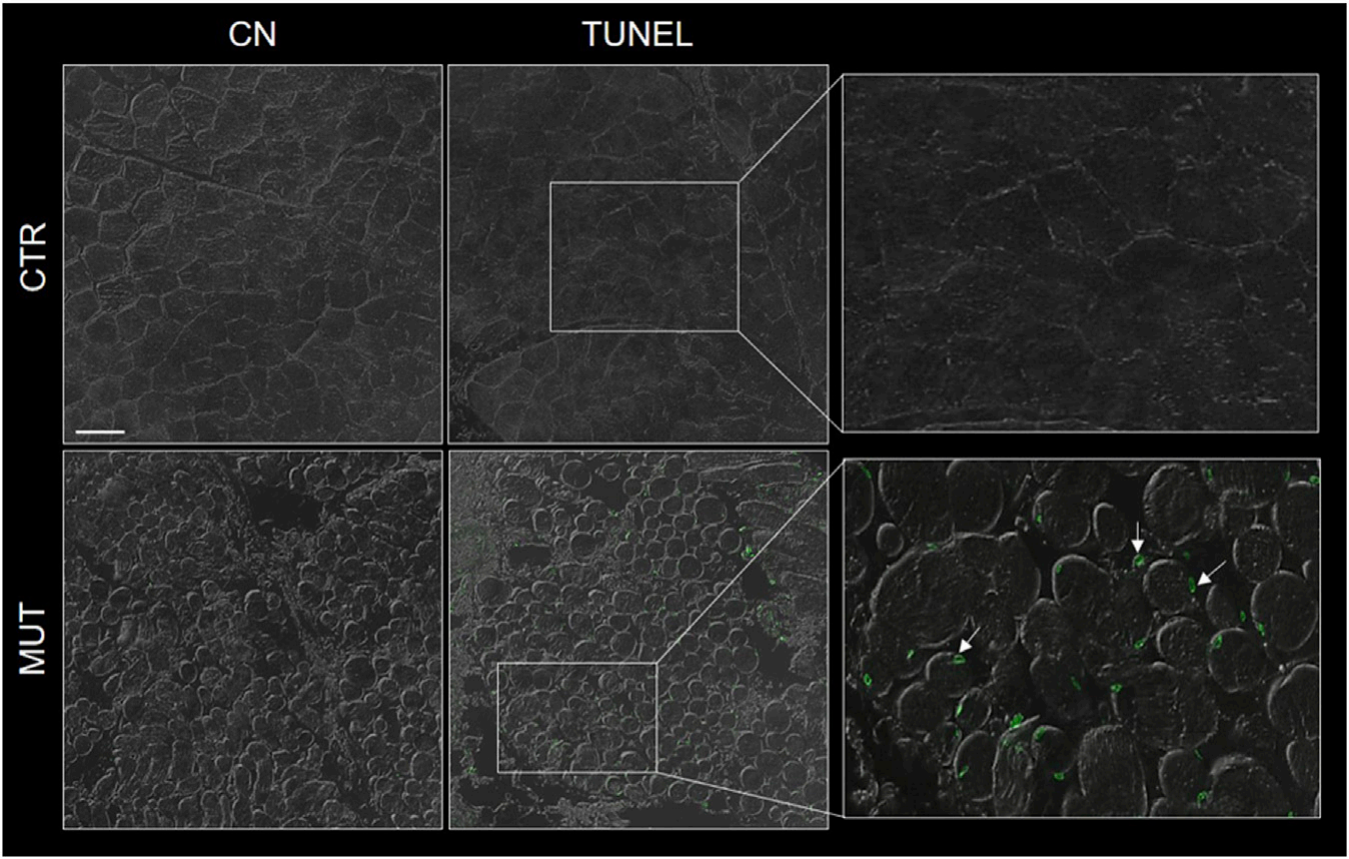
Representative image of TUNEL assay of MUT and CTR muscles. DNA-strand breaks (green spots were more abundant in the MUT (white arrows in the panel with enlargement to the far right, bottom) an in the CTR muscle. CN, negative control for MUT and CTR muscles without TdT enzyme. Bar = 100 μm.

MUT muscle showed many more apoptotic fibers than CTR muscle by the TUNEL assay (Figure 2).

### 3.3 CCT5 Presence, Distribution, and Colocalization With CCT1

**FIGURE 3:**
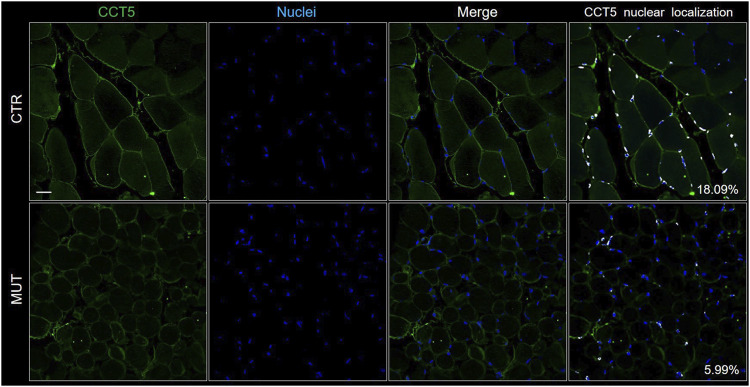
Localization of CCT5 on CTR and MUT muscles. Immunofluorescence was done with FITC conjugated anti-CCT5 antibody (green); nuclei were stained withe Hoechst (blue). Nuclear localization of CCT5 (white spots) in CTR muscle (18.09%) was more frequent than in MUT muscle (5.99%). Bar = 25 μm.

In the CTR muscle, CCT5 was present in the sarcolemma, cytoplasm, and within the nuclei (Figure 3, CTR panels) but its signal was slighter in the cytoplasm, nuclei, and, but not as much, in the sarcolemma of MUT muscle fibers (Figure 3, MUT panels). In the MUT muscle, CCT5 was detected in the extracellular space (intercellular matrix) (Figure 3, MUT panels).

**FIGURE 4:**
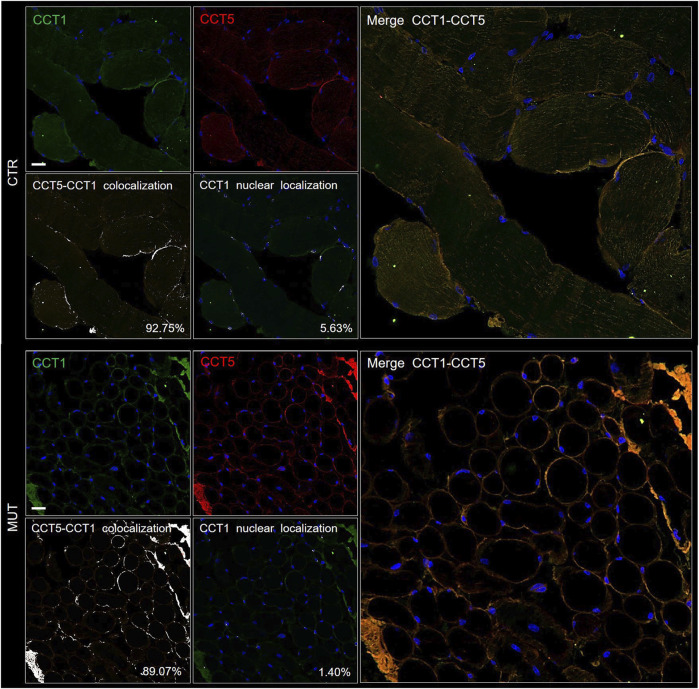
Double immunofluorescence for CCT5 and CCT1 subunits on CTR (top set of five panels) and MUT (bottom set of five panels) muscles. CTR and MUT muscles were stained with FITC conjugated anti-CCT1 antibody (green) and with TRITC conjugated anti-CCT5 antibody (red). Nuclei were stained with probe Hoechst (blue). Colocalization of CCT5 and CCT1 in CTR muscle (92.75%) and in MUT (89.07%) muscle appeared as white spots (see the two panels at the bottom left corner of the CTR and MUT sets of panels). Bar = 25 μm. Nuclear localization of CCT1 in CTR (5.63%) and in MUT (1.40%) muscles appeared as white spots (see the two middle panels at the bottom of the CTR and MUT series of panels with the legend “CCT1 nuclear colocalization”). Bar = 25 μm.

To determine if the CCT5 subunit in the MUT muscle was associated to other components of its chaperoning team, i.e., the CCT oligomer, colocalization experiments were performed, using double immunofluorescence with antibodies against CCT5 and CCT1. The two subunits colocalized in the MUT and CTR muscles (Figure 4). The results for CCT5 confirm the localization shown in Figure 3: it appears, here together with CCT1, in the sarcolemma, cytoplasm and nuclei of CTR muscle, whereas in the MUT muscle the two subunits appear mostly in sarcolemma and the extracellular matrix (Figure 4). The lower signal of CCT1 in nuclei of MUT muscle closely parallel the low signal of CCT5, suggesting these subunits tend to associate with each other in MUT muscle as well.

### 3.4 Actin and Myosin

**FIGURE 5:**
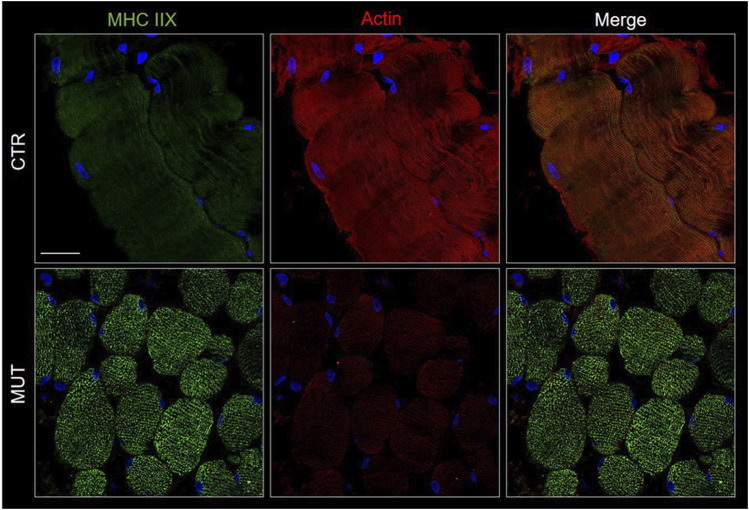
Detection of myosin (MHC IIX) with FITC-conjugated antibody (green) and actin with TRICT-conjugated antibody (red) in CTR and MUT muscles. Myosin positivity was higher in the MUT than in the CTR muscle whereas actin positivity showed the reverse pattern being higher in the CTR muscle. Also, the distribution patterns of the two proteins differed in the CTR and MUT muscles and colocalization was absent. Nuclei were stained with probe Hoechst 33342 (blue). Bar = 25 µm.

Actin and myosin distribution patterns in MUT and CTR muscles were similar, but myosin signal was slightly increased, and that of actin was decreased in MUT as compared with CTR muscle (Figure 5).

### 3.5 Desmin

**FIGURE 6:**
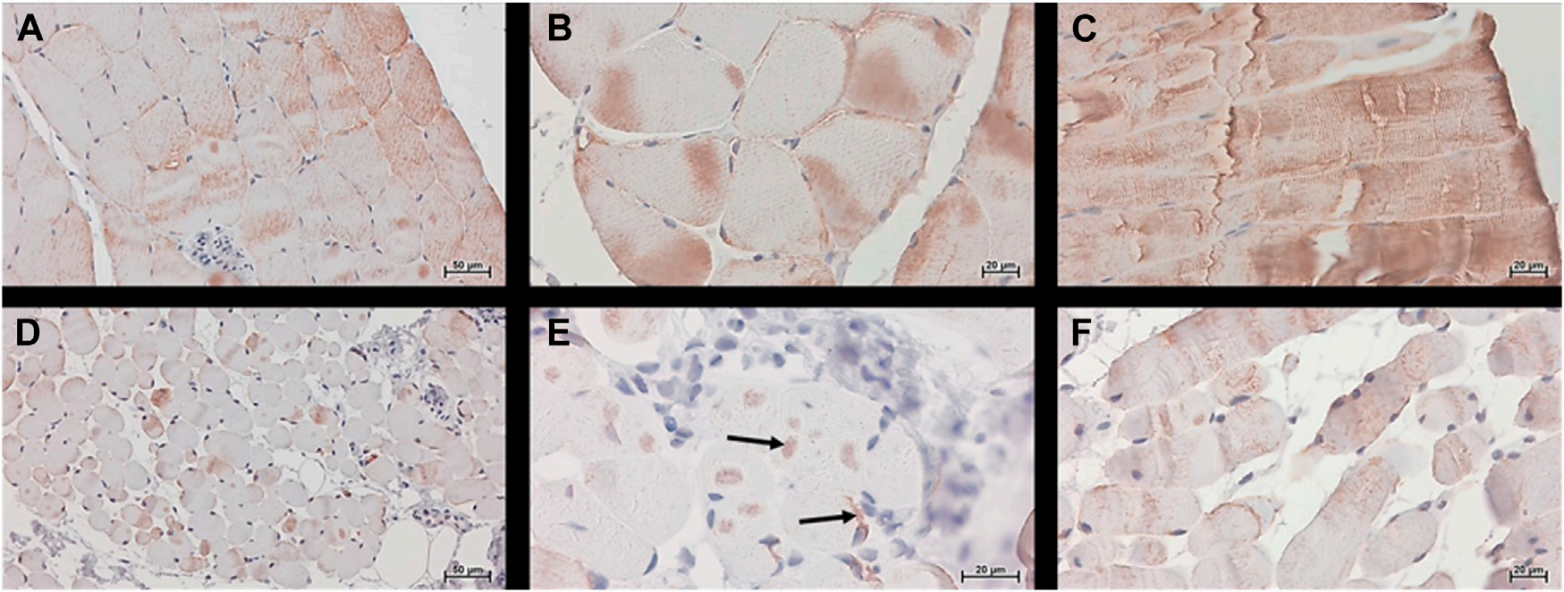
Desmin was detected histochemically, using the Histostain®-Plus third Gen IHC Detection Kit, which revealed desmin (brown color) in CTR (panels **A–C**) and MUT (panels **D–F**) muscles. **(A,D)** Magnification ×200; scale bar 50 μm; **(B,C,E,F)** magnification ×400; scale bar 20 µm. Desmin was present in the cytoplasm and in the sarcoplasmic membrane of the CTR muscle. In MUT muscle, desmin appeared at lower levels than in the CTR muscle in the sarcoplasm and as aggregates in paracentral and peripheral locations (examples indicated by arrows on panel **E**). The banding pattern typical of normal muscle in longitudinal section (panel **C**) was absent in the muscle from the patient (panel **F**).

Desmin (muscle-specific type III intermediate filament) is a muscle-specific protein, normally localized in the sarcolemma, Z-disc, and nuclear membrane, that maintains the sarcomere structure, linking the sarcolemma, mitochondria, lysosomes, and nucleus. In CTR muscle, desmin was visible in the sarcolemma and cytoplasm with the expected banding pattern in longitudinal sections (Figures 6A–C panels). In MUT muscle the presence of desmin appeared lower than in CTR muscle and its distribution pattern was different (Figures 6D–F panels). The desmin banding pattern was absent in MUT muscle, and aggregates containing this protein were present at paracentral and peripheral locations within the muscle fibers (Figure 6E).

### 3.6 CCT5, Alpha-B-Crystallin, Hsp90, and Desmin

**FIGURE 7:**
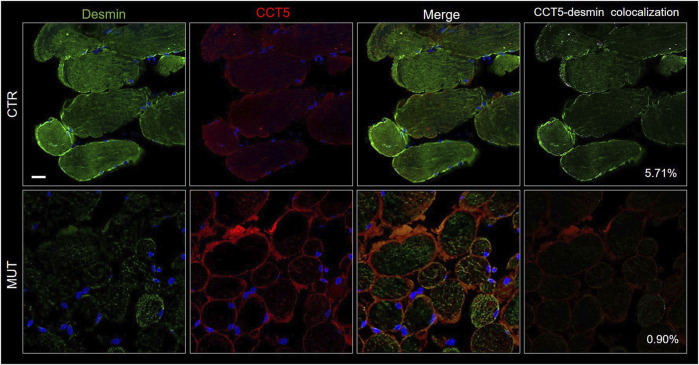
Double immunofluorescence with FITC-conjugated antibody for desmin (green) and with TRITC conjugated anti-CCT5 antibody (red) in CTR and MUT muscles. Solid desmin positivity at the costamer level occurred in CTR muscle while it appeared as disorganized dots in the MUT muscle. CCT5 and desmin colocalization was reduced in MUT muscle (0.90%) as compared with the CTR muscle (5.71%). Nuclei were stained with probe Hoechst 33342 (blue). Bar = 25 µm.

**FIGURE 8:**
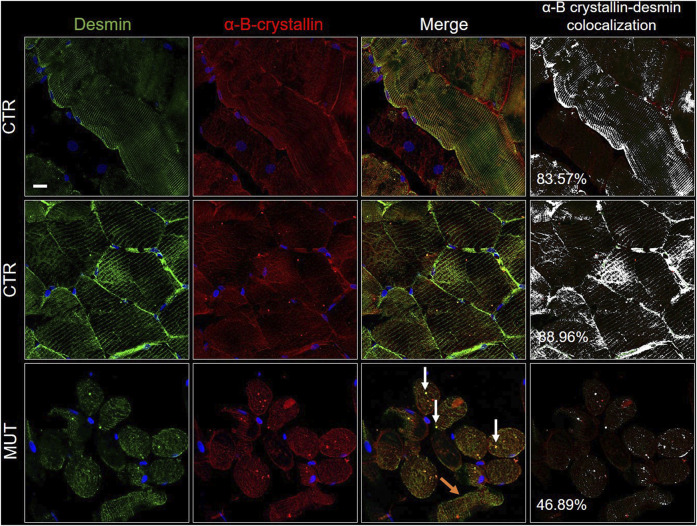
Double immunofluorescence with FITC-conjugated antibody for desmin (green) and with TRITC conjugated anti-alpha-B-crystallin antibody (red) in CTR and MUT muscles. Alpha-B-crystallin positivity in the sarcolemma and sarcoplasm of CTR muscle fibers appeared in the longitudinal (top row of CTR panels) and the transversal sections (middle row of CTR panels); this positivity was weaker in the MUT muscle fibers (bottom row, MUT panels). Fiber disorganization was evident (orange arrows on the MUT panel to the right, one before the last or “merge” panel). On this same “merge” panel, white arrows point to examples of alpha-B-crystallin and desmin aggregates. Alpha-B-crystallin and desmin colocalization was higher in CTR muscle (longitudinal section, 83.57%; transversal section, 88.96%) than in MUT muscle (for example, only 46.89% in MUT muscle transversal section, as show in this figure). Nuclei were stained with probe Hoechst 33342 (blue). Bar = 25 µm.

**FIGURE 9:**
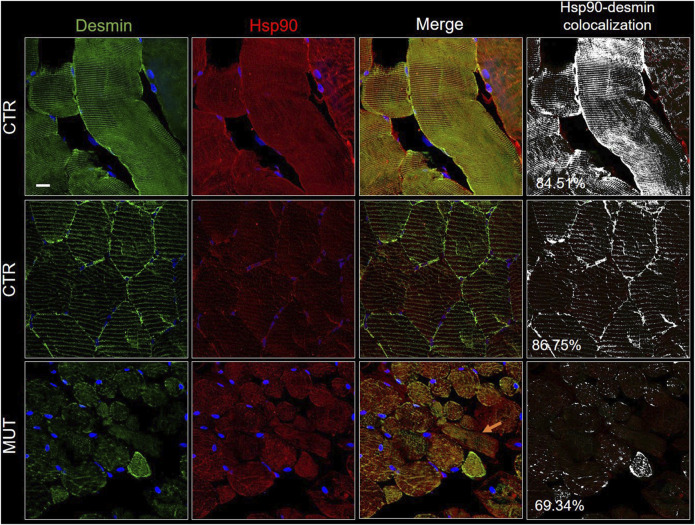
Double immunofluorescence with FITC-conjugated antibody for desmin (green) and with TRITC conjugated anti-Hsp90 antibody (red) in CTR and MUT muscles. Hsp90 positivity was well organized in longitudinal and transversal sections of the CTR muscle, but in contrast, it was disrupted and weaker in the MUT muscle (see for example the MUT muscle longitudinal fiber indicated by the orange arrow on the MUT “Merge” panel). Hsp90 and desmin colocalization was high in CTR muscle (longitudinal section, 84.51%; transversal section, 86.75%) by comparison with the MUT muscle (for example, it was 69.34% in MUT muscle transversal section, as shown in this figure). Nuclei were stained with probe Hoechst 33342 (blue). Bar = 25 µm.

The CS components CCT5, alpha-B-crystallin, and Hsp90, the latter two normally associated at the level of the Z-discs, and desmin were assessed to determine presence and distribution. We performed double immunofluorescence for: 1) desmin and CCT5 subunit (Figure 7B); desmin and alpha-B-crystallin (Figure 8C) desmin andHsp90 (Figure 9). Desmin was homogeneously distributed in CTR muscle along the Z-discs, seen as horizontal striations and with a striated pattern in cross- and longitudinal sections, respectively (green fluorescence in CTR panels of Figures 7–9). In MUT muscle, signal of desmin appeared slighter than in CTR muscle and appeared as irregular or trabecular patterns, and as dots (green fluorescence in MUT panels of Figures 7–9). In some fibers the desmin-labelled sarcolemma was not visible.

The cytoplasm of MUT muscle fibers showed little if any signal for CCT5 subunit, but this signal was present in the sarcolemma and the intercellular matrix, as also illustrated in Figure 3. Almost no colocalization of CCT5 with desmin was observed in the extracellular matrix of affected tissue (Figure 7, rightmost MUT panel).

Alpha-B-crystallin was uniformly distributed in CTR muscle showing a striated pattern in longitudinal and cross sections (Figure 8). In contrast, in MUT muscle, alpha-B-crystallin signal was milder, appearing as a light band in longitudinal sections and as precipatetes with desmin in the sarcoplasm (white arrows in MUT merge panel of Figure 8). Likewise, colocalization of alpha-B-crystallin and desmin was reduced in half in MUT muscle as compared with CTR muscle.

Hsp90 was distributed throughout the cytosol of the CTR muscle fibers in a well-organized pattern in longitudinal and cross sections (Figure 9, CTR panels). In the MUT muscle, Hsp90 was present but appeared in an irregular pattern somewhat like that of desmin (Figure 9, MUT panels). Hsp90 and desmin colocalization was reduced in the MUT muscle as compared with the CTR muscle.

### 3.7 In Silico Analysis of the CCT5 Mutant Protein

The histopathological abnormalities found in the MUT muscle suggested poor protein homeostasis, which can, as a working hypothesis, be attributed to a defective CCT complex carrying the mutant CCT5 subunit. Therefore, we investigated the impact of the mutation on the CCT5 protein molecule. We used bioinformatics as we did in a previous work with another mutant of CCT5 because, in that work, we detected molecular abnormalities caused by the mutation that accurately predicted the abnormalities of the CCT5 subunit molecular properties and functions that were also demonstrated by wet lab experiments (Min et al., 2014; Spigolon et al., 2017; Macario and Conway de Macario, 2020).

#### 3.7.1 CCT5 Structure

The properties and functions of the CCT5 subunit and its mutant can be visualized better by looking at the alignment of the amino acid sequences of the wild type and mutant molecules, showing the structural domains named equatorial, intermediate, and apical, and the subdivision of the former two into N-terminal and C-terminal segments, Supplementary Figure S2. Other comparative analyses of the two molecules are facilitated by keeping in mind the details shown in the alignment figure.

#### 3.7.2 Comparison of Molecular Models

**FIGURE 10:**
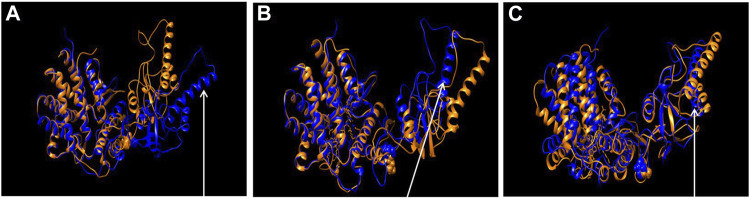
Comparison of wild type and the mutant CCT5 using molecular models. Superposition of the most probable conformations obtained by molecular dynamics simulations of wild type CCT5 (orange) and the mutant Leu224Val CCT5 (blue), nucleotide free **(A)**, ATP-bound **(B)**, and ADP-bound **(C)**. Spheres in the intermediate domain correspond to Leucine (orange, wild type) and Valine (blue, mutation) at position 224. Noteworthy in panel **(A)** is the greater opening of the mutant protein, especially at the level of the α9 helix, compared to the wild-type protein (arrow). In panel **(B)**, the α9 helix of the mutant CCT5 is more closed than in the wild type (arrow), in contrast with the situation in the nucleotide-free conditions, shown in panel **(A)**. In panel **(C)**, under the ADP-bound conditions, the mutant protein shows the lid still more closed that in the wild type (arrow).

To learn about the impact of the mutation on the CCT5 apical domain configuration previously reported for the Leu224Val mutant (Antona et al., 2020), we superposed wild type and mutant molecular models under three conditions, nucleotide free, ATP bound, and ADP bound, Figure 10. It was confirmed that the mutant protein differs from the wild type, particularly at the level of the apical domain.

#### 3.7.3 Radius of Gyration

**FIGURE 11:**
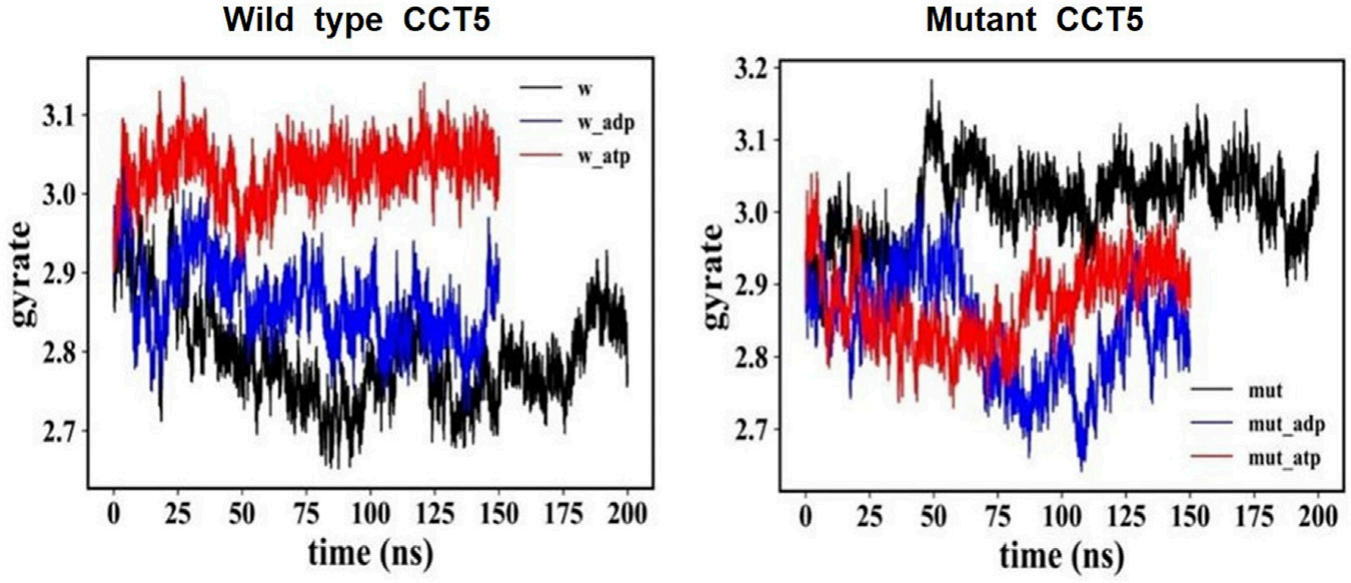
Distribution of atomic masses in the wild type and mutant CCT5 by radius of gyration analysis. Comparison of the radius of gyration of wild type (left panel) and mutant (right panel) CCT5 subunits as a function of time was done under the three conditions studied: nucleotide free (black), ATP bound (red), and ADP bound (blue).

We used the radius of gyration (RG) versus time method to determine if the distribution of atomic masses in the mutant was different from that of the wild-type protein under three conditions: nucleotide free, ATP bound, and ADP bound (Figure 11). All trends of the mutant protein showed different RG values than the wild-type counterparts. For instance, the nucleotide-free mutant (Figure 11, right panel, black) had a trend opposite to that of the wild-type nucleotide-free protein, with increasing values, 2.9-3.2 RG for the mutant and decreasing values, 2.9-2.7 RG, for the wild-type molecule (Figure 11, left panel, black).

#### 3.7.4 Root Mean Square Deviation (RMSD)

**FIGURE 12:**
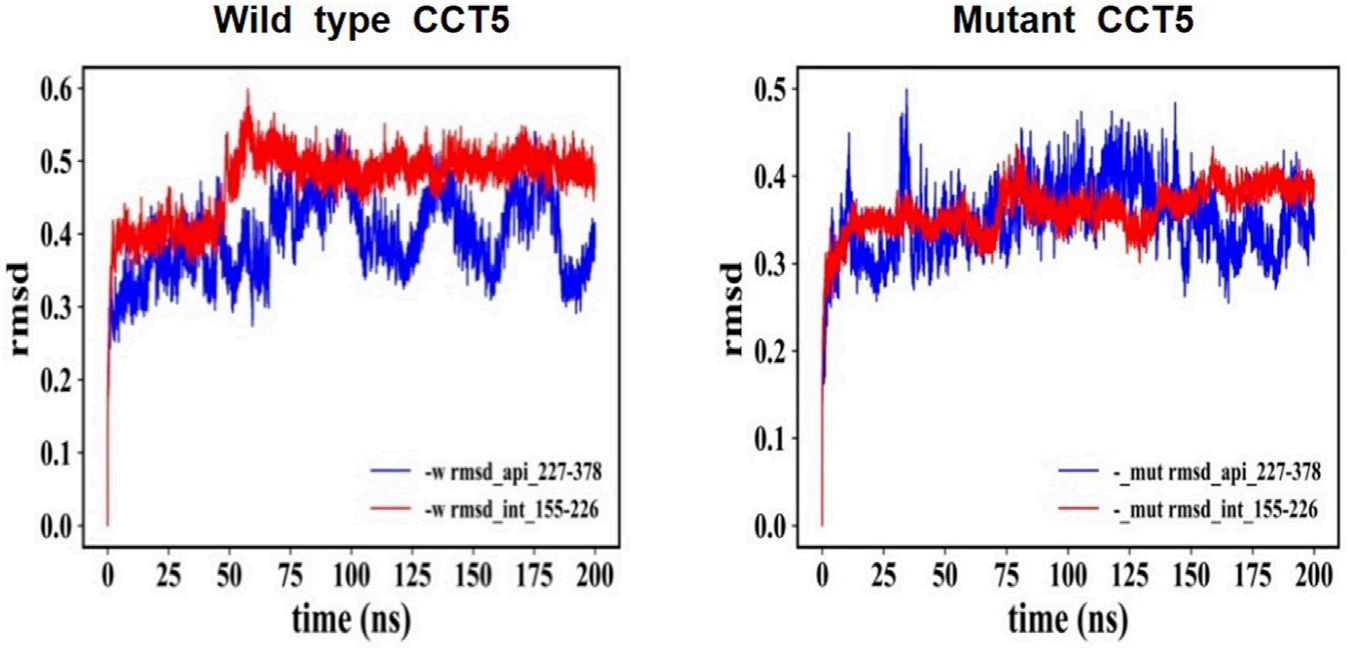
RMSD: Comparison of nucleotide-free CCT5, wild type vs. mutant. RMSD of the entire molecules, wild type and mutant CCT5, were determined, and the outputs were examined in various ways. Here, we present the comparison of the parts of the outputs pertaining to the N-terminal segment of the intermediate domain and to the apical domain, which illustrates the difference between the wild type and the mutant molecules. Left panel: Wild type molecule apical domain, blue; N-terminal segment of intermediate domain, red. Right panel: Mutant molecule apical domain, blue; N-terminal segment of intermediate domain, red. The RMSD values reveal differences between the two molecules and instability of the apical domain of the mutant. Similar trends were shown when ADP and ATP bound molecules were examined, although in the ATP-bound molecules the differences in instability of the mutant with regard to the wild-type molecule were less marked (data not shown).

The CCT5 molecule, like the other CCT subunits consists of three domains, apical, equatorial, and intermediate and while the latter two are composed of two separate segments of the protein’s amino sequence, the apical domain consists of single segment (Supplementary Figure S2). Molecular dynamics simulations were carried out to determine if there were differences between the domain segments of the mutant and the wild type proteins nucleotide free, and ATP- or ADP bound. The RSMD analysis showed substantial differences between the two molecules, particularly at the level of the apical domain (Figure 12).

#### 3.7.5 Heat Maps

**FIGURE 13:**
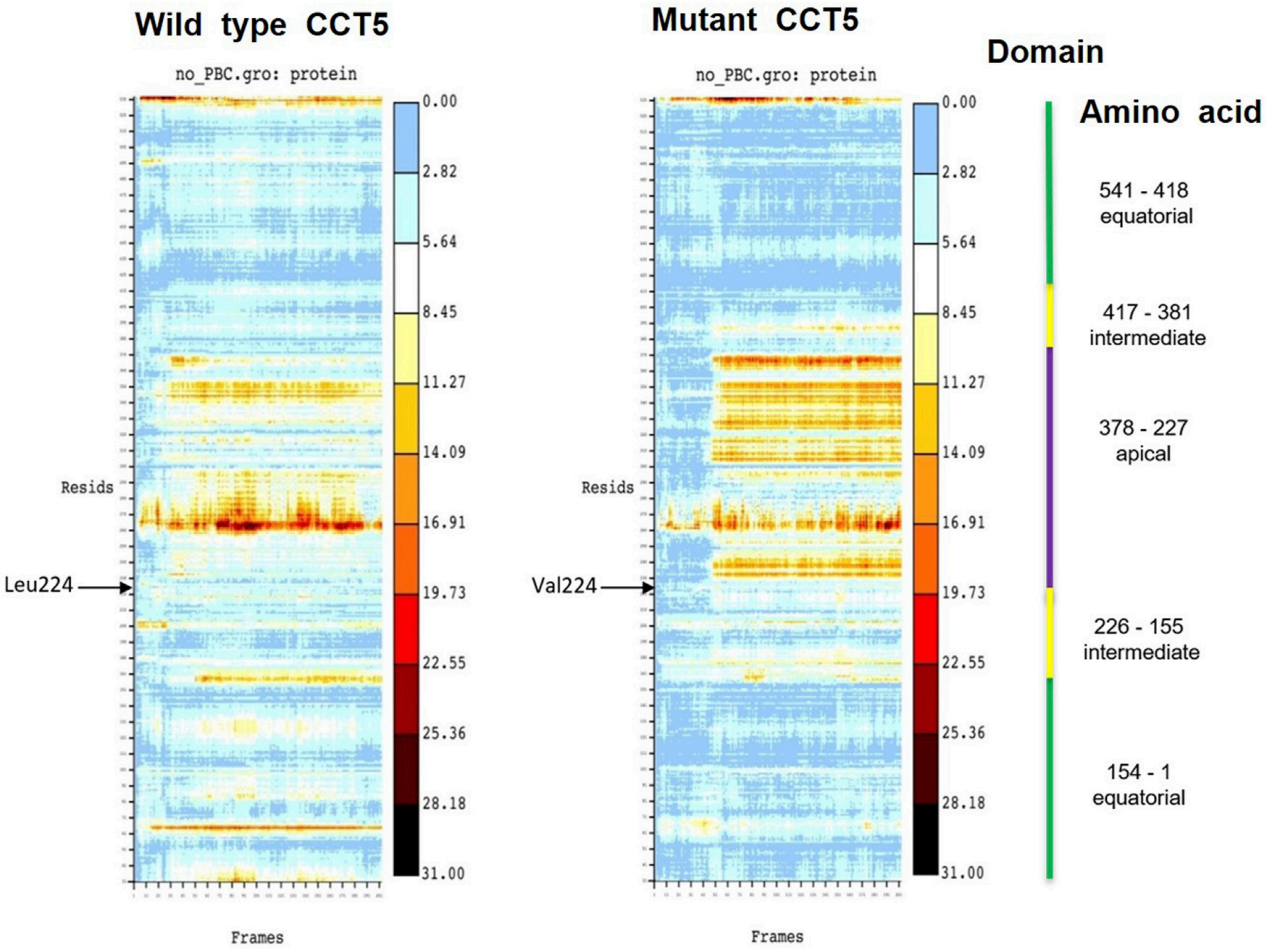
Heat maps of wild type and mutant nucleotide-free CCT5 subunits. In the heat map of the wild type subunit a wider area of heat is observed in the region between the amino acids (Resids) 260 and 270 (at 11.27 Å) and between amino acids 230 and 250 (at 16.91 Å). These two regions are within the apical domain (indicated by a violet vertical line on far the right). In the mutant subunit a wider heat area is observed in a region between amino acids 230 and 370 (between 19.73 and 16.91 Å), which is in the intermediate domain, beginning very close to position 224 in the N-terminal segment of the intermediate domain, where the point mutation occurs (indicated by the black arrow Val224), and it spans the entire apical domain.

To identify the amino acids within each domain involved in the molecular conformational changes observed by molecular dynamics simulations, we resorted to heat maps. The heat maps show the RMSD value in Angstrӧm unit (Å, in *y*-axis) of each individual amino acid residue during simulations (frame in ns, in *x*-axis). Collectively, these analyses revealed instability of the apical domain of the mutant nucleotide-free protein (Figure 13).

## 4 Discussion

Many diseases of the central and peripheral nervous systems and of skeletal muscle are proteinopathies, namely, the central pathogenic-diagnostic molecule is a defective protein, which in most instances is the product of a mutant gene, thus the diseases are by definition genetic, and for the most part hereditary (Jameson et al., 2018). When the abnormal protein belongs to the chaperone system (CS), the disease can be classified as a chaperonopathy (Macario and Conway de Macario, 2005). In this report, we deal with a disease that according to clinical signs and symptoms is a genetic, early onset, distal motor neuropathy, associated to a mutation in a chaperone gene, the subunit CCT5 of the chaperone complex CCT (Antona et al., 2020). We report the histopathological characteristics of the skeletal muscle of the patient, focusing on details that in principle might be the result of deficient protein homeostasis caused by inadequate chaperoning.

Information on the tissue abnormalities found in chaperonopathies and the molecular mechanisms underpinning these abnormalities, including the role of the defective chaperones, is scarce and badly needed (Macario and Conway de Macario, 2020). There is some information on the histopathological manifestations of myopathies, including one associated to a chaperonopathy, but little is available on specific aspects of protein homeostasis that might be affected by faulty chaperoning (Ruggieri et al., 2015; Cassandrini et al., 2017).

It is noteworthy that, in our patient, there is considerable disruption of the skeletal muscle architecture coinciding with abnormalities of desmin in the absence of cardiac manifestations of disease. Desmin is essential to maintain the structure of not only the skeletal but also the cardiac muscle. Diverse mutants of the desmin gene are associated with myopathy and cardiopathy (Brodehl et al., 2018). Alterations of desmin expression have been described in conditions of muscle atrophy induced by disuse and aging (Marzuca-Nassr et al., 2017). Also, the size of nuclei in muscle tissue may increase when desmin is functionally impaired (Heffler et al., 2020). If the desmin abnormalities in our patient are the result of deficient chaperoning by CCT, one may ask why the myocardium does not seem to be affected. An explanation could be derived from understanding the CS physiology. The CS components are distributed in all cells and tissues but assembled in groups of interacting molecules that are distinctive for each cell and tissue type, thus while the CCT chaperoning team may be present in the cytosol of all cell and tissue types its interacting partners (i.e., other teams to form networks, co-chaperones, chaperone co-factors, and chaperone interactors and receptors (Macario and Conway de Macario, 2020) are not the same in all locations. Therefore, a defective CCT team bearing a pathogenic mutant may show a range of capabilities depending on the cell type. This may explain the observation that desmin is considerably affected in the skeletal muscle while it is not in the cardiac muscle since the patient does not show clinical manifestations of heart disease. Another explanation could be that CCT does not participate directly in the folding of desmin, or in any of the steps leading to its maturation to become a functional protein in the appropriate cell locale but plays an indirect role. For example, CCT could be key for the folding and maturation of a protein, e.g., an enzyme, required by desmin production and migration to its physiological locations in skeletal but not in the cardiac muscle. If the CCT complex with the mutant CCT5 subunit fails in its chaperoning functions regarding that protein, desmin would be altered.

In the last few years, it has been found that, differently from other subunits, CCT5 (and in a less competent manner also the CCT4 subunit) has the ability to form homo-oligomeric complexes (micro-complex and single ring), supporting the hypothesis that single CCT5 homo-oligomeric rings may be the base assembly units for the formation of the functional hetero-oligomeric CCT hexadecamer (Sergeeva et al., 2019; Liu et al., 2021). A certain functional ability of the mutant CCT5 in our patient is suggested by its ability to form part of the CCT team, i.e., the CCT hexadecamer. This is suggested by the finding that CCT5 colocalized with CCT1, another component of the CCT team, in which these two subunits are not in direct contact (Leitner et al., 2012). Thus, one may conclude that if the mutant CCT5 colocalizes with CCT1 it must do so indirectly, through contact with the other subunits; and this would indicate that the mutant subunit must be competent to join the other subunits, form the octameric ring, and the full hexadecameric chaperoning machine. This is in line with the fact that the mutation affects primarily the apical domain of CCT5, namely, the domain involved in substrate recognition and chaperoning chamber closing and opening, but not in the formation of rings and hexadecamers (Lopez et al., 2015). Thus, one can expect a certain level of chaperoning ability from the CCT complex, only curtailed by insufficient capability for substrate recognition and handling and depending on the cell type’s proteome, which is different for each cell type. Consequently, one can expect diverse kinds and levels of failure of protein homeostasis in different cell and tissue types. To further complicate the landscape of deficient protein homeostasis in the whole organism stands the diversity of possible substrates for CCT, which include a variety of proteins: structural (Yam et al., 2008), regulatory of cell cycle and apoptosis (Willison, 2018a; 2018b), and others involved in neural tissue development and degeneration (Chánez-Cárdenas and Vázquez-Contreras, 2012). Substrate selection and binding depend on specific amino acid sequences on the client protein (Willison, 2018a; 2018b) and on the translational context (Yam et al., 2008). In different tissue types (with distinct proteomes), and under different conditions (e.g., normal vs stress condition), CCT can bind different substrates. This adds to the diversity of effects that a defective CCT complex may cause in various tissues, for example, striated skeletal and cardiac muscles, and nervous tissue and skeletal muscle.

Another finding that also indicates that the chaperonin complex with the mutant CCT5 subunit can function, at least to some extent and for a reduced range of substrates, is that the signal for actin, a canonical substrate for CCT (Berger et al., 2018; Willison, 2018a, 2018b), was only slightly affected in the patient’s muscle. It can therefore be inferred that while actin, normally the substrate for which CCT has high avidity, is still recognized by the defective chaperone, whereas other less preferred substrates are not.

The CCT complex, and the Hsp90 and alpha-B-crystallin chaperones are at the Z-disc of healthy skeletal muscle and are believed to play key roles in the development and maintenance of this structure (Roh et al., 2015; Unger et al., 2017; Berger et al., 2018). Our experiments demonstrate that all three components are affected in MUT muscle, which would impair the functionality of the Z-disc; Z-lines would not anchor the contractile filaments and, in turn, desmin would not transmit the force of contraction to sarcolemma, hampering the shortening of muscle fibers. Alpha-B-crystallin is a desmin chaperone but which of the two proteins is primarily damaged is to be investigated. Hsp90 and alpha-B-crystallin genetic variants are related to hereditary myopathies (Reilich et al., 2010; Unger et al., 2017). Many fibers of skeletal muscle tissue from the patient were in apoptosis, but TUNEL assay does not allow us to differentiate the classical apoptosis, from denervation and necrosis, and further investigations are required. However, a skeletal muscle histomorphology due to inactivity and comparable to the one described here is not reported in literature, whereas protein aggregation and reduction of myofibrils in zebrafish *cct5*-mutant model was accompanied be severe decrease of skeletal muscle-dependent force generation (Berger et al., 2018). Therefore, it is highly likely that failure of CCT entails defects involving alpha-B crystallin and Hsp90, which is not surprising considering the physiological interactions known to occur between different members of the CS.

The *in silico* analyses of the mutant CCT5 in comparison with the wild type counterpart, demonstrated changes in the mutant, most evident in the apical domain, which suggest that the chaperonin is defective and could be the cause of some of the abnormalities observed in our patient.

The CCT complex fold diverse substrates, typically actin and tubulin, which differ in structure. This suggests that substrates for CCT do not have to have a specific amino acid signature for recognition and binding by the chaperonin but must show other features for the purpose. For instance, matching tridimensional conformations in the chaperone and the substrate could be involved with participation of multiple weak interactions. In this regard, the radius of gyration and RMSD analyses revealed in the mutant molecule an energy disturbance, suggesting altered folding with opposite masses distribution and a different molecular equilibrium as compared with the wild type CCT5 subunit. The apical domain of the mutant subunit showed a major deviation of atoms in comparison with the wild-type counterpart, most notably under nucleotide-free conditions but also appreciable under ATP-bound conditions. Helix-10 of the apical domain (amino acids _309_EANHLLLQ_316_) and the proximal loop region (amino acids _234_FSHPQMPK_241_) are involved in substrate-binding (Pereira et al., 2017). Beyond the substrate-binding region, the extended α-helix (helix-9) of the apical domain functions as a built-in lid to close the CCT hexadecamer’s cavity and is linked to an apical loop (amino acids _259_KPKTKHK_265_), which creates a highly charged region probably involved in substrate interaction (Pereira et al., 2017). Heat maps of the mutant CCT5 subunit confirmed the instability of the apical domain affecting helix-10, proximal loop, and helix-9 position in absence of nucleotide. It is, therefore, possible that the chaperoning functions of the CCT hexadecamer with the mutant CCT subunit are deficient with compromise of substrate recognition, binding, and conveyance. Thus, it is likely that at least some of the abnormalities observed in the striated muscle of the patient are the consequence of deficient chaperoning.

Here we report for the first time the histomorphology of a genetic chaperonopathy in the skeletal muscle tissue. We believe that, taking into account the difficulty to reproduce the complexity of a tissue *in vitro*, our results provide the indispensable platform for designing and launching the experiments necessary for elucidating the molecular mechanisms underpinning the reported tissue abnormalities.

## Data Availability

The original contributions presented in the study are included in the article/Supplementary Material, further inquiries can be directed to the corresponding author.
