# Supplementary material for: Muscle Histopathological Abnormalities in a Patient With a CCT5 Mutation Predicted to Affect the Apical Domain of the Chaperonin Subunit
Source: Front Mol Biosci. 2022 Jun 2;9:887336. doi: 10.3389/fmolb.2022.887336 (PMC9201415; doi:10.3389/fmolb.2022.887336)
Supplement: Supplementary file 1 [file DataSheet1.PDF]

**Supplementary Material**

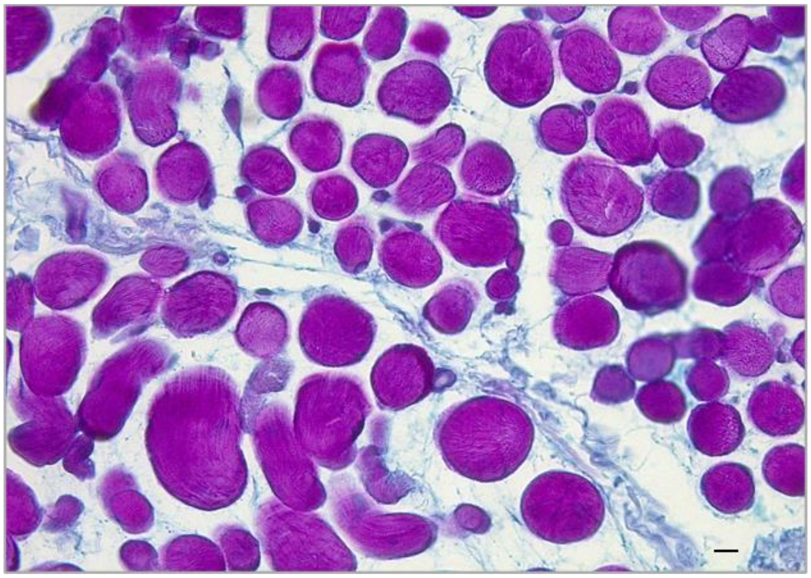

**Figure S1 -** Alcian-Pas staining of MUT muscle. In the transversal section shown, muscle fibers appear round and deeply stained (dark violet). Bar=100µm.

**CLUSTAL ALIGNMENT OF AMINO ACID SEQUENCES**

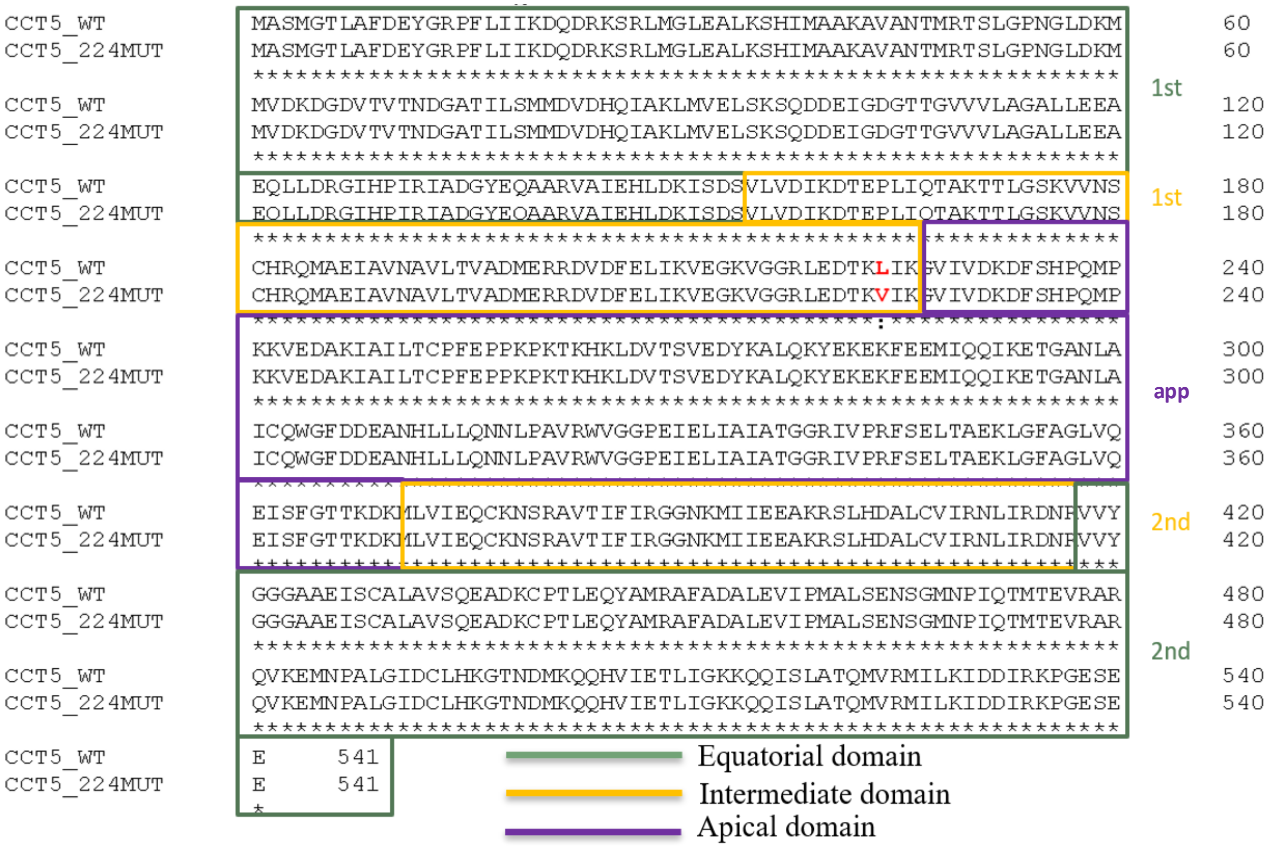


**Figure S2 -** CCT5 structural domains. Clustal alignment of wild type and mutant CCT5 amino acid sequences (CCT5_WT and CCT5_224MUT respectively). Leu (WT) and Val (MUT) in position 224 are in red color. The colored boxes show: **a**) the equatorial domain (green) 1st or N-terminal segment, from amino acid 1 to 154, and 2nd or C-terminal segment, from amino acid 418 to 541; **b**) the intermediate domain (yellow) 1st or N-terminal segment, from amino acid 155 to 226 (in this segment occurs the pathogenic mutation Leu224Val), and 2nd or C-terminal segment, from amino acid 381 to 417; and **c**) the apical domain (violet), from amino acid 227 to 378.
